# Declining lake ice in response to climate change can impact spending for local communities

**DOI:** 10.1371/journal.pone.0299937

**Published:** 2024-07-05

**Authors:** Alessandro Filazzola, Mohammad Arshad Imrit, Andrew Fleck, Richard Iestyn Woolway, Sapna Sharma

**Affiliations:** 1 Department of Biology, York University, Toronto, Ontario, Canada; 2 Apex Resource Management Solutions, Ottawa, Ontario, Canada; 3 Department of Mathematics and Statistics, York University, Toronto, Ontario, Canada; 4 School of Ocean Sciences, Bangor University, Menai Bridge, Anglesey, Wales; KFUPM: King Fahd University of Petroleum & Minerals, SAUDI ARABIA

## Abstract

Lake ice is an important socio-economic resource that is threatened by climate change. The cover and duration of lake ice are expected to decline as air temperatures warm in the coming decades, disrupting a previously reliable source of income for many activities dependent on lake ice. The economic consequences of climate-induced lake ice loss remain unexplored, creating a significant research gap. The purpose of this study was to quantify the monetary spending associated with lake ice and how climate change may impact that value. Using a series of General Circulation Models (GCMs), greenhouse gas emissions scenarios, and models for lake ice cover, we predicted changes in lake ice by the end of the 21st century for the Northern Hemisphere. We also synthesized examples of spending associated with lake ice activities and discussed the potential implications expected with declining ice cover. We found that lake ice will decrease in area by 44,000–177,000 km^2^ and shorten in duration by 13–43 days by 2100. Using 31 examples of revenue from lake ice, we found that lake ice generates spending of over USD 2.04 billion to local communities and economies. We also found that countries predicted to experience the greatest ice loss by the end of the century are those that currently have the largest GDP, highest greenhouse gas emissions, and are most dependent on freshwater withdrawal. Our findings confirm predicted losses in lake ice that are expected because of climate change and quantify some of the potential consequences for local communities. Here we highlight lake ice as another casualty of human-caused climate change that will have profound socio-economic implications.

## Introduction

Natural ecosystems provide a suite of services in support of human well-being and the global economy. Freshwater lakes regularly provide a resource for fishing, potable water, and recreational activities. In winter, the ice on freshwater lakes is an important cultural and economic resource for many communities. For example, the loss of ice can delay the construction of winter ice roads, which can affect the transportation of goods to remote communities [1, 2]; delay or cancel skating, ice-fishing, and other recreational tournaments [3]; alter hunting seasons for traditional foods for Indigenous communities, including whales, seals, and caribou [4]; and result in increased winter drownings in warmer winters with less stable ice cover [5]. The monetary value of ecosystem services is rarely quantified, leaving the costs of global environmental stressors largely unknown. This is especially important given the higher cost of inaction relative to climate change mitigation [6, 7]. In the few cases where costs have been estimated, the monetary values are significant, such as invasive species negatively affecting the global economy by 26.8 billion USD annually [8] or lake eutrophication expected to cost 81 trillion USD by 2050 [9]. Water resources have already begun to display evidence of an economic decline from climate change [10], highlighting the urgency of evaluating the financial value associated with freshwater ecosystems.

Global warming caused by human-induced climate change is already having pronounced biophysical implications globally [11]. Many regions around the world have identified droughts or warming that exceeds long-term averages in climate monitoring [e.g., 12–14]. Winter is a rapidly warming season [15, 16] threatening the ice cover of freshwater lakes and the services they provide. Climate change is causing the widespread loss of lake ice including shorter seasons [17], more frequent ice-free winters [18], and in extreme cases, the permanent loss of lake ice cover [19]. One of the first physical lake responses to climatic warming in many lakes is the loss of ice cover due to the tightly linked relationship between winter air temperature and freshwater freezing [i.e., 20–22]. The loss of winter ice cover portends the loss of a northern cultural identity, which has social, cultural, and health values that cannot be quantified [23]. As climate change reduces lake ice cover and duration, the spending generated from these winter activities is at risk of being lost. Although we have a good understanding of future lake ice patterns under climate change, the financial implications of lake ice declines have never been attributed.

Here, we conducted the first exploration of the spending generated by lake ice and the potential declines anticipated from climate change. Using process-based numerical models of lake ice cover across the Northern Hemisphere until the end of the 21st century, we quantified changes in ice cover, ice duration, and revenue of lake ice activities to ask the following three questions: 1) How is the duration and cover of lake ice in the Northern Hemisphere expected to change over the coming decades?, 2) What is the estimated spending generated from lake ice?, and 3) What are the socio-economic implications of decreasing lake ice? The findings from this study will offer valuable insights for informing policies to support local communities as they experience shifts in revenue from changes in lake ice duration.

## Methods

### Data acquisition

We conducted a modelling study to forecast the effects of climate change on lake ice duration and quantify the financial implications for local communities. Lake ice projections used in this study were simulated using an ensemble of process-based lake models, which contributed to the Inter-Sectoral Impact Model Intercomparison Project phase 2b (ISIMIP2b) Lake Sector [24]. These models include the Community Land Model version 4.5 (CLM4.5) [25], the Arctic Lake Biogeochemistry Model (ALBM) [26, 27], SIMSTRAT-UoG [28], and LAKE [29]. Each lake model was driven by climate data from four General Circulation Models (GCMs) which contributed to phase 5 of the Coupled Model Intercomparison Project (CMIP5), GFDL-ESM2M, HadGEM2-ES, IPSL-CM5A-LR and MIROC5, after bias-adjustment to the EWEMBI reference dataset [30, 31]. These models are well documented and are a culmination of multiple research teams estimating the biophysical responses of climate change [32]. Detailed methodology and adjustments of all ISIMIP models, including the Global Lake Sector, are available online (https://www.isimip.org/impactmodels). These models have also been previously compared to *in situ* observations and found to have significant agreement between the two [33].

Historical simulations (1901–2005) represent a historical climate, whereas future projections (2006–2099) consider three Representative Concentration Pathways (RCPs) representing low greenhouse gas emissions (RCP 2.6), moderate emissions (RCP 6.0), and high emissions (RCP 8.5) [34]. Climate input data to the lake models included projections of air temperatures at 2 m, wind speed at 10 m, surface solar and thermal radiation, relative humidity, total precipitation, surface air pressure, and snowfall, all of which were available at a daily resolution. Following the ISIMIP2b global lake sector protocol, each of these lake models were used to simulate daily lake ice cover at a 0.5°-by-0.5° grid resolution, based on the mean depth and surface area of all known lakes within a 0.5° grid. Notably, for each 0.5° longitude-latitude grid, the lake models simulated the ice cover of a “representative lake”, with its size and depth determined by the distribution of all known lakes within that grid. The locations, depths, and grid-scale fractions of lakes within each 0.5° grid were determined by the Global Lake Data Base version 1, originally provided at a 30 arc-second resolution [35–37].

Post-processing of daily modeled lake ice cover was performed to attain homogenized ice onset, break-up, and duration values following Grant et al., [33]. Specifically, ice-cover indices were calculated with hydrological years, defined as year-long periods that contain ice onset or break-up dates. For ice onset and break-up dates, we select the October to September and September to August hydrological years, respectively. Ice duration was computed as the sum of all ice cover days across the October to September hydrological year. Furthermore, we converted the ice off and ice on dates to be centered around January 1st as 0. Hence, dates before January 1st are negative and dates after January 1st are positive.

In total, we had 16 unique lake-climate models represented by combinations of four GCMs and four lake models under three different RCPs to estimate the timing of ice-on (n = 45) and ice-off (n = 45). We excluded three model combinations with anomalously high values: HadGEM2-ES-CLM4.5-RCP8.5, MIROC5-LAKE-RCP8.5, and GFDL-ESM2M-LAKE-RCP8.5.

### Estimates of lake ice duration and area

We calculated the mean ice duration, change in the area of ice cover, and change in the duration of ice cover, for each pixel across all of the lake models and climate scenarios. We then calculated the average ice duration and total area of ice cover per year across the Northern Hemisphere for each of the lake models and climate scenarios. To quantify the relative change in ice patterns, we calculated the contemporary annual average (between 1970 and 1999) of ice area and ice duration and subtracted each yearly value from this historic value. Furthermore, we calculated a 31-year rolling mean (function *rollapply*, package *zoo* in the R computing language) to observe future patterns of ice area controlling for interannual variability. We selected a 31-year rolling average to capture variability in natural climate cycles such as the El Niño-Southern Oscillation, Pacific Decadal Oscillation, and solar cycles.

### Estimating the spending generated by lake ice

We used two types of searches to find examples of spending on activities associated with lake ice. In the first search, we chose countries in the Northern Hemisphere. Using the search terms (lake ice) AND (revenue OR econom* OR spend*) and the name of a country in the Northern Hemisphere (e.g., Russia, Canada) to identify articles and sources that contained financial values associated with lake ice. Articles were chosen if they explicitly mentioned lake ice and had a monetary value attached to them. We searched using these terms in both Google and Google Scholar through over 20 pages until no other documents could be found in English. We included all articles that had economic data within them (n = 31). Most of these articles were newspaper articles or government documents and none were peer-reviewed articles.

In the second search, we used the above search terms along with the names of several specific lakes that we identified in the first search as having some form of monetary revenue. We acknowledge that our search used only English search terms, potentially neglecting articles written in the languages of the target countries. Thus, we supplemented our searches by asking limnologists across North America, Europe, and Asia from the Global Lakes Ecological Observatory Network (GLEON) for additional examples.

The monetary values identified in each article were presented as a number or in a table. We extracted these values into a spreadsheet and converted all foreign currency into United States Dollar (USD) using the January 2022 exchange rate. To project the revenue changes in lake ice activities, we maintained the reported monetary value constant, factoring in only the variations in lake ice duration. This involved multiplying the monetary value by the percentage change in ice duration for each year until 2099, specific to the corresponding year and geographic area outlined in the respective study.

We obtained country-level information for countries with lake ice in the Northern Hemisphere from the World Bank Indicator Database (https://data.worldbank.org/indicator). For all countries with lake ice, we obtained information on Gross Domestic Product in 2019 (USD), total annual freshwater withdrawals in 2017 (billion cubic meters), and greenhouse gas emissions in 2016 (metric tons per capita). For the political borders of each country, we used the administrative boundaries provided by the Database of Global Administrative Areas (https://gadm.org/). In total, 50 countries were identified to have lake ice in the Northern Hemisphere. For each country, we calculated the total area of lake ice across all pixels found within the political boundaries. The area of lake ice for each country was average across all lake models and climate scenarios but separated for each year and each of the three RCP scenarios. We determined the area of ice lost for each country by subtracting the mean area observed between 1970–1999 from 2070–2099.

### Statistical analysis

To determine if changes in the surface area of lake ice were associated with the socio-economic characteristics of countries, we fit linear models with the difference in area of lake ice (between the two timeframes) as the response variable. As predictors, we crossed the RCPs with each of the four socio-economic variables: GDP, annual freshwater withdrawal, and greenhouse gas emissions. Both the change in the area of lake ice and each socio-economic variable was log-transformed to meet assumptions of normality and account for the order of magnitude differences in the variables of some countries. All analyses were conducted in R Version 4.1.2 [38] using packages *raster* [39], *ncdf4* [40], *zoo* [41], and *tidyverse* [42]. All code used for the estimation of lake ice patterns, economic calculations, and statistical analyses are available at https://afilazzola.github.io/IceVariability/.

## Results

### Climate change effects on lake ice

**Fig 1:**
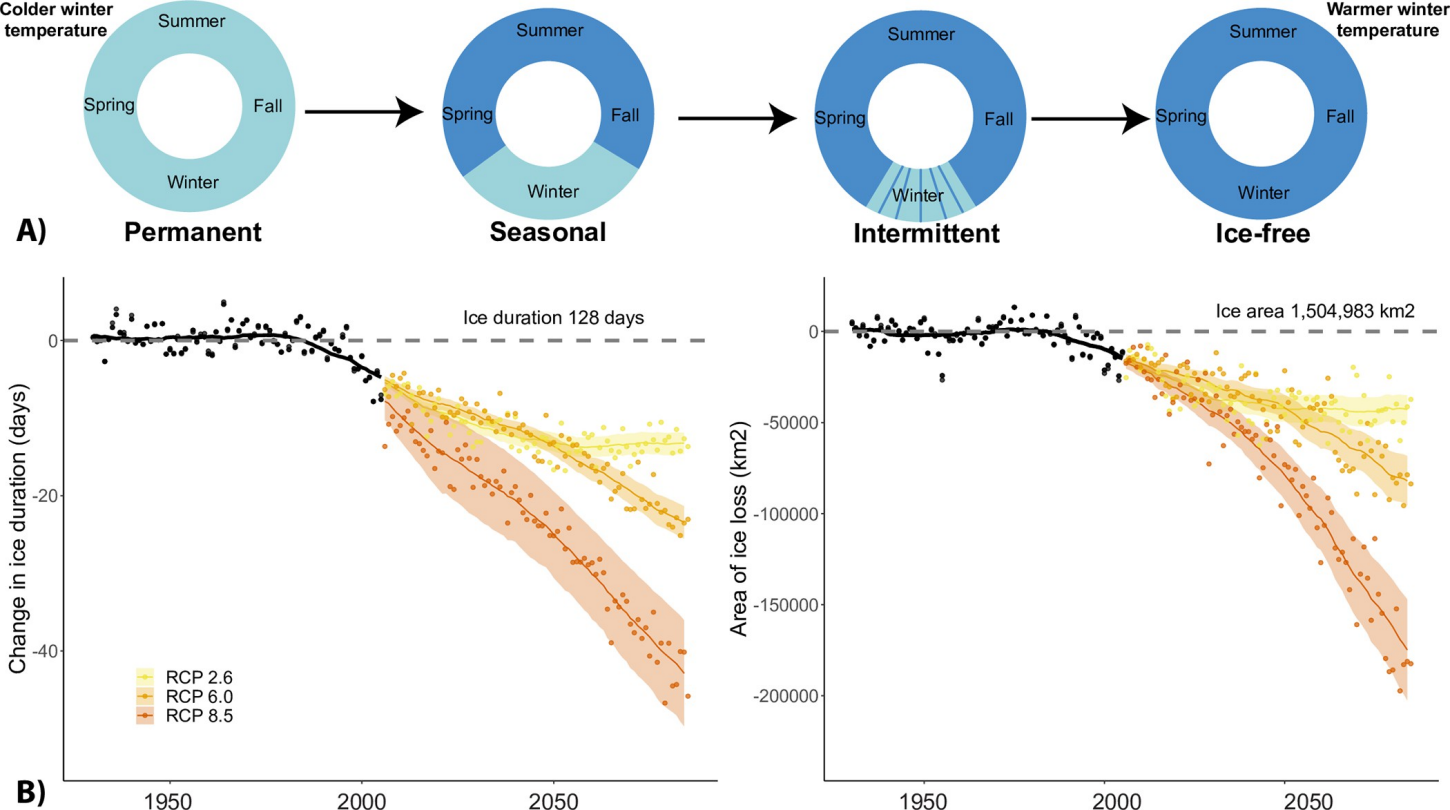
Patterns of lake ice cover are changing throughout the Northern Hemisphere. Lakes can be categorized as having permanent ice cover, seasonal ice cover (e.g., during winter), intermittent ice cover with multiple freeze-thaw events, and permanently ice-free (Panel A). With warmer temperatures, lakes transition from permanent ice cover to seasonal ice cover, and from seasonal ice cover to permanently ice free. Under a modest increase in greenhouse gas emissions (i.e., RCP 2.6) the duration of lake ice will decline by approximately 10 days. However, if greenhouse gas emissions rise substantially (RCP 8.5), on average the duration of lake ice will decrease by more than 40 days (Panel B). Consequently, the area of lake ice will decline as some lakes transition to completely ice-free. Depending on the greenhouse gas emissions, we predict a loss of between 50,000 and 200,000 km^2^ of ice cover. Lines represent mean model fit and standard error band represents the uncertainty associated among the different GCMs and lake models used to estimate ice phenology.

Under climate change, lakes are transitioning to shorter ice durations or not freezing entirely. Lakes can display four types of ice phenologies including permanently frozen, seasonally frozen (e.g., winter only), intermittently frozen (i.e., freeze-thaw cycles), or completely ice-free (Fig 1A). Our models suggest that currently there are over 1.5 million km^2^ of lake area that freezes annually with an average ice duration of 128 days (Fig 1B). We predict that by the end of the century, we will lose between 43,568 ± 2,012 km^2^ (mean 2070–2099 ± SE; RCP 2.6), 82,199 ± 2,587 km^2^ (RCP 6.0), and 176,796 ± 5,618 km^2^ (RCP 8.5) of lake ice cover (Fig 1B). We also predict that the duration of ice cover will decrease, on average, between 13.2 ± 0.3 days (mean 2070–2099 ± SE; RCP 2.6), 23.5 ± 0.5 days (RCP 6.0), and 43.2 ± 0.9 days (RCP 8.5) (Fig 1B).

### Local revenue from lake ice

**Fig 2:**
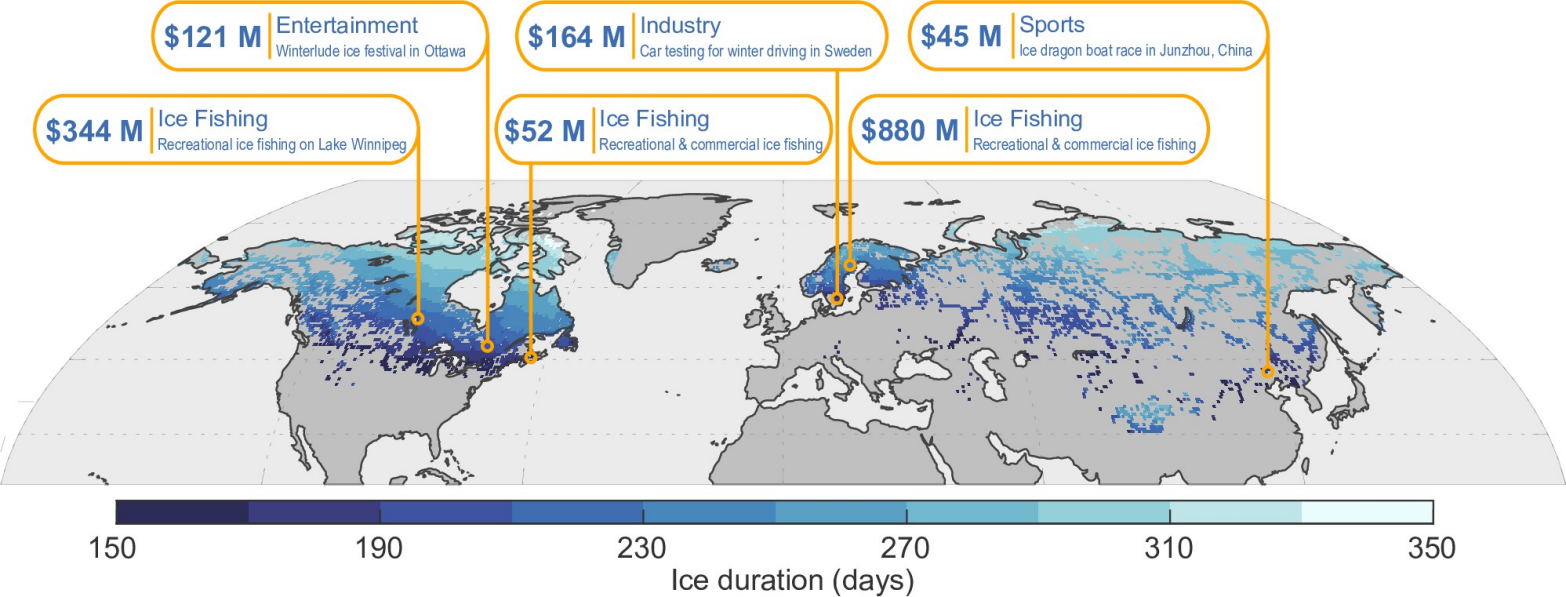
Lake ice is an important resource globally. Across the Northern Hemisphere, ice covers freshwater lakes on average ~150 days per year and up to 365 days per year at higher latitudes. The monetary value of lake ice is diverse including fishing, industrial manufacturing, recreational activities, and winter festivals. Inset in the figure are some examples of the revenue ($USD) generated by activities dependent on lake ice. Ice cover duration values shown in the top panel are based on the average simulations across the lake-climate model ensemble during the contemporary period (1970–1999). Map was generated using the data from GADM (https://gadm.org/) under a CC BY license with permission from the GADM organization, original copyright 2018–2022.

Lake ice is responsible for considerable spending on activities through multiple streams including winter festivals, car testing in winter conditions, ice fishing, and sports competitions (Fig 2, Table 1). Ice fishing is a commonly reported source generating money for local communities adjacent to frozen lakes and is responsible for USD 1.30 billion of spending generated annually in our examples (Table 1). For example, Lake Winnipeg in Canada can generate as much as USD 350 million annually from ice fishing alone [43]. This spending includes direct spending by the anglers, but also indirect benefits to GDP, salaries to local residents, and tax revenue for the regional government [43]. Similarly, the Swedish town of *Arjeplog* is a popular test site for winter driving by automakers and generates USD 164 million annually [44]. With the predictions of lake ice duration to decline by between 10.3% (RCP 2.6) and 33.7% (RCP 8.5), we could expect changes in spending habits that equate to millions each year, cumulating to billions by the end of the century.

**Table 1:** Examples of event spending from lake ice activities for local communities. For additional details including specific coordinates, original currency, and data source see S1 Table.

Activity Description	Country	Regions/Lakes	Event Spending ($USD)	People attending
Ice Fishing	Sweden	Many	$880,000,000	
Recreational Ice fishing	Canada	Lake Winnipeg	$344,093,275	> 100,000
Ice Fishing	USA	Many	$178,000,000	
Car Winter Testing	Sweden	Many	$163,800,000	> 3,000
Winterlude	Canada	City of Ottawa	$121,471,499	600,000
Ice Fishing	Canada	Province of Ontario	$57,946,000	
Ice Fishing	Canada	Province of Nova Scotia	$51,730,000	
Ice Dragon Boat race	China	City of Jinzhou	$45,000,000	4.47 million
Winter angling activities	USA	State of Minnesota	$36,500,000	
Ice canoe race, Bonhomme Winter Festival	Canada	Quebec City	$30,325,900	1,000,000
Ice fishing culture tourism festival	China	Chagan Lake	$29,000,000	100,000
Ice Fishing	Canada	Bay of Quinte	$24,830,839	300,000
Ice Fishing	Canada	Lake Simcoe	$23,175,450	
Horse Racing	Switzerland	Lake St. Moritz	$20,400,000	10,000
Recreational Ice Fishing	USA	Devil’s lake	$20,000,000	
Ice Fishing	Canada	City of Ste-Anne-de-la-Perade	$4,788,300	100,000
Lake Louise Winterstart World Cup	Canada	Lake Louise	$2,979,648	7,700 (> 100 million global viewers)
Devils Lake Volunteer Fire Department Ice Fishing Derby	USA	Devil’s lake	$1,300,000	> 5,000
Ice boat championship	Canada	City of Kingston	$186,214	
Wawa Ice Fishing Derby	Canada	Wawa Lake	$144,846	1,300
Winter Festival of Speed	Canada	Lac la Biche	$124,152	> 100
Ice dragon boat (international)	Canada	City of Ottawa	$79,805	25,000
Alberta 55-plus winter games	Canada	Cold Lake	$57,194	900
Brainerd Jaycess Ice Fishing Extravaganza	USA	Gull Lake	$13,400	1.15 million
International Eelpout Festival in Walker	USA	Leech Lake	$12,000	
Sylvan Lake polar bear dip	Canada	Sylvan Lake	$10,526	
Fishing For Ducks Contest	USA	Mille Lacs Lake	$5,000	150,000

### Socio-economic implications of decreasing lake ice

**Fig 3:**
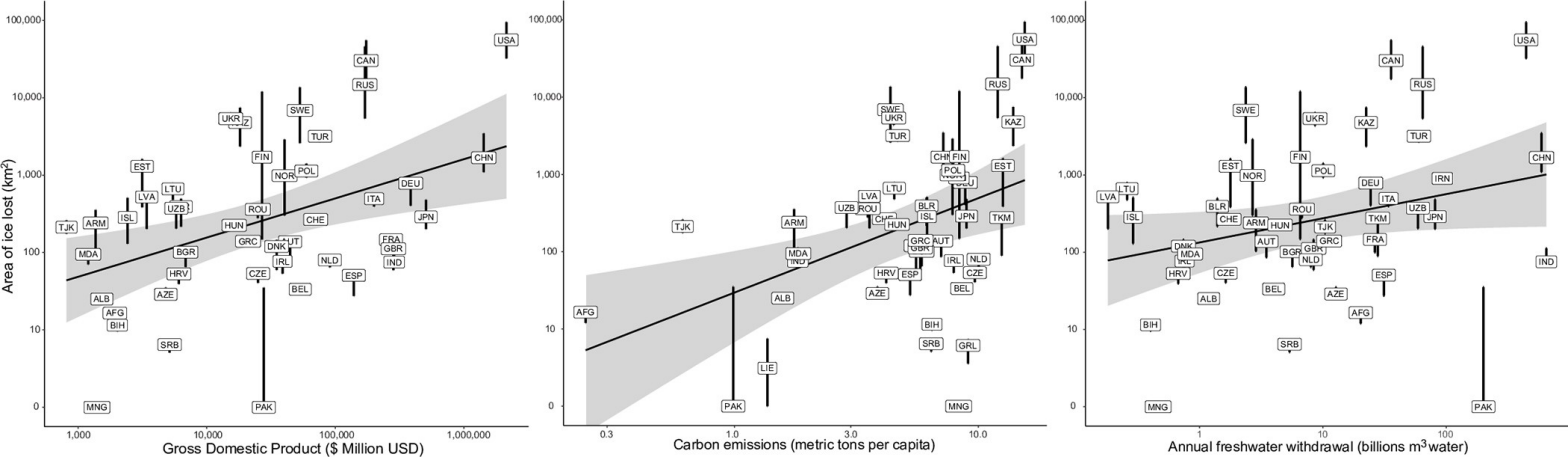
Patterns of lake ice loss for countries in the Northern Hemisphere and association with the socio-economic metrics including gross domestic product ($USD), carbon emissions (per capita), and annual freshwater withdrawal. Countries with the highest gross domestic product, largest carbon emissions, and greatest withdrawal of freshwater are all expected to also have the greatest loss of lake ice area. Three letters correspond to a country code (S2 Table) with the line above and below representing minimum (RCP 2.6) and maximum (RCP 8.5) ice loss.

The countries anticipated to have the greatest ice loss by the end of this century are the countries currently with the largest GDP (Fig 3), who also tend to spend the most money on activities that use lake ice (Fig 2 and Table 1). We found that the greater loss of lake ice per country correlated with increased greenhouse gas emissions (F_137_ = 13.5, p < 0.0001; Fig 3), and higher GDP (F_131_ = 22.6, p < 0.0001; Fig 3), regardless of greenhouse gas emission scenario (all p > 0.05). We also found that countries with the greatest area of ice loss are the most dependent on freshwater for commercial and residential purposes (F_137_ = 16.3, p < 0.001; Fig 3).

## Discussion

Lake ice is a valuable resource that is expected to decline in the coming decades. Previous research has identified lake ice as threatened by climate change both through modelling [19, 45] and *in situ* observations [3, 17, 18, 46]. We estimated that in the Northern Hemisphere, lake ice will become on average shorter by 13–43 days and have between 44,000 and 177,000 less km^2^ of ice cover, contributing to shifts in spending by the end of the century. Lake ice is strongly dependent on winter air temperatures to freeze, where increases in mean air temperature or extreme warm events can lead to shorter freeze durations [17, 47], intermittent ice cover [45, 48], ice-free years [22, 49], or in the most extreme case, the permanent loss of ice cover [19]. Lakes that are large and shallow have been identified as the most vulnerable to lake ice loss from climate change because of the high surface area with the air, sensitivity to ice breakup from wind, and lower heat latency from less water volume [46, 50, 51]. Understanding the mechanisms through which lake ice is affected by climate change is particularly important to identify the specific lakes and associated activities that will be most impacted by climate change. Our findings reinforce the widespread loss of lake ice because of climate change, highlighting the decline of an important socio-economic resource.

### The value of lake ice activities

Shorter periods of ice cover are likely related to a shorter window for activities (e.g., fishing season is shorter, fewer opportunities for events such as ice festivals or skating races) decreasing the possible revenue generated. In our 31 identified examples, we found that lake ice contributes over USD 2 billion in spending for local communities and economies that will decline in the coming decades. Our examples included lakes with a wide range of characteristics, suggesting even small lakes play an integral economic role in the communities they are situated in. For communities dependent on lake ice activities, the loss of millions in local spending can disrupt the economic stability of these regions. Shorter ice cover duration could result in event cancellations and a complete loss of revenue (i.e., 100% decline) for some activities that occur in specific time windows (e.g., winter festivals, sports competitions). For example, a skating race on Lake Mälaren in Sweden generated income for a remote Nordic region but was cancelled indefinitely in 2018 because of unpredictable ice conditions [3]. These communities will be forced to gamble running events at the risk of having no lake ice or unsafe ice that may not be thick enough to sustain human activities [5]. For events that continue, organizers may incur higher costs. What has historically been decades of reliable revenue for communities near lakes will decline and become unreliable as climate change continues to shorten the duration of ice cover.

The specific economic examples provided are based on historical values in recent years but are by no means an exhaustive list. There are more examples of economic consequences of declining lake ice that have not been quantified, such as the value of ice roads for winter transportation [1], recreational activities (cross-country skiing, snowshoeing, skating) [52], and other winter festivals. For example, winter sports in the United States have been estimated to contribute USD 11.3 billion to the national economy [53], which includes downhill skiing and snowboarding, but also activities likely to require lake ice, such as backcountry skiing, snowshoeing, and snowmobiling. These latter activities have been rapidly increasing in popularity, with backcountry skiing and snowshoeing equipment sales exceeding USD 50 million during the 2015–2016 winter [54]. Additionally, indirect costs include all the business-to-business related revenue, such as purchasing local goods and services, and induced effects include increasing wages and staff that further facilitate the local economy and generate revenue [55]. In the United States, every dollar spent directly on winter sports activities can generate an additional $1.55 from indirect and induced effects [53]. More importantly, although there could be instances of economic benefits associated with declining ice cover, it comes at the cost of environmental degradation which cannot be economically quantified. Therefore, the spending associated with lake ice activities is significantly larger than the USD 2.04 billion we have collated. While this value cannot be easily extrapolated to represent a global value, our results present the initial steps in quantifying the direct and indirect monetary impacts of lake ice loss.

With climate change causing the loss of lake ice, local communities may shift spending towards open-water activities. Instead of ice-based activities, individuals may choose to participate in water recreational activities, such as boating, swimming, and open-water fishing. In these cases, income spending is not necessarily lost for the local communities because local businesses can reinvest their offerings to cater to the changing recreational preferences. For example, marinas and boat rental companies may see increased demand for their services that will consequently mitigate the loss of customer base from ice-activities for local hotels and restaurants. The shift towards water-based activities can also lead to increased investment in infrastructure necessary to support these activities, such as boat ramps, docks, and hospitality amenities. Previous work has identified potential economic benefits of climate change, such as increased agriculture production [56], greater recreation activities in the arctic [57, 58], and benefits for the wind energy industry [59]. For instance, reduced sea ice has been suggested to increase the activity of vessels for fishing, cruises, or cargo [60, 61], and these patterns will likely also be reflected in large freshwater lakes (e.g., the Great Lakes). However, the benefits of climate change are complex and likely to disrupt the stability of many communities adjacent to lakes that previously froze. While ice-based activities could be moved to lakes that are continuing to freeze (e.g., higher latitudes or altitudes), the infrastructure might not exist in the new locations to support either the activities or the residents. Geo-political borders may further complicate the movement of ice-based activities as regional or national governments have different requirements, incentives, and infrastructure. Adaptations will be required for communities to continue to deliver activities, such as replacing snowmobiles with boats [62]. Governments will likely need to support communities undergoing transition in spending from loss of lake ice especially since these communities are already receiving less income from declining cover. The uncertainty for lakes around “will it, won’t it” freeze could also prevent investment into these communities as lakes may not freeze consistently or be safe enough to support ice-activities, but freeze often enough that lakes in warmer climates may be more appealing for open-water activities. Ultimately, the shift away from ice-based activities presents an opportunity for new growth and development in water-based activities, but the loss of stable revenue and transitionary costs will diminish some of the potential benefits.

The loss of ice has profound implications for water quality both within the winter season and across seasons [63]. For example, under-ice chlorophyll concentrations and primary production may increase with decreased ice duration [64, 65]. Earlier ice-off contributes to a longer open-water season, earlier, longer, and stronger stratification [14] and warmer water temperatures [66, 67], with consequences to declining hypolimnetic oxygen concentrations [68], reduced oxythermal habitat for freshwater fishes [69], amongst a broad suite of implications on freshwater quality and availability [2]. Thus, while our study explored only the spending impacts of declining ice in the months where the lake is expected to freeze (e.g., winter), there are likely costs that will extend into the summer months.

### Socio-economic implications of lake ice loss

For many residents, their relationship with lake ice extends well beyond a source of income and is an integral component of their cultural identity (Magnuson and Lathrop 2014). People have used lake ice for ceremonial activities, artistic inspiration, education, and recreation [For a review, see 3]. There is a connection between people and lake ice that extends beyond economic value to include childhood memories at winter ice festivals, an integral part of the diet (i.e., ice fishing) for some communities [70], and a significant cultural value [3, 18]. For indigenous communities, the loss lake ice directly impacts food security, creating greater uncertainty around the location, abundance, and composition of wildlife [4, 57, 62]. For example, loss of lake ice can impact reindeer husbandry as the caribou alter their migration patterns [71]. Ice-based activities play an important role in the social fabric of these communities, providing opportunities for socializing, bonding, and intergenerational knowledge sharing (Magnuson and Lathrop 2014). The loss of lake ice thus not only threatens the financial stability of northern communities but also their cultural identity.

Anthropogenically driven climate change has already begun impacting the physical characteristics of our planet [72, 73] and lake ice is no exception [74, 75]. We observed significant differences in the outcomes of lake ice based on the projected greenhouse gas emission scenarios (Fig 2). For high latitude countries, the differences between RCP scenarios translate to 10, 100, or even 1000 times more area of ice being lost. The burdens of climate change are typically associated with countries with lower GDPs, and also those that currently emit the least greenhouse gas emissions, making these countries least able to mitigate future negative impacts [76, 77]. However, lake ice represents one of the few instances where the countries impacted by climate change are the most capable of mitigating change. Since it is the countries with the highest emissions that have the most to lose (Fig 3), our findings should therefore further encourage action to mitigate emissions.

### Limitations

While modelling studies are powerful, allowing the synthesis of patterns at a global scale, we recognize there are some limitations in our methodology. Firstly, we used gridded simulations that are averaged patterns over 0.5° longitude-latitude grids where individual lakes will likely deviate either more or less in duration. This is further complicated by the limited availability of examples of lake ice revenue, restricting a comprehensive analysis of consequences from different activities or geographic regions. The ISIMIP models also do not provide any information on snow, which is an important factor influencing ice dynamics and may impact lake ice activities. Finally, our study is based on stacked uncertainty, where the uncertainty in climate models, compounded by the dependence of lake models on these climate models, reduces the confidence in predictability. We discuss this stacked uncertainty and the interaction with discount rates in our S1 File. Our study represents an initial step to estimate the financial impacts of lake ice loss in a global context, but these limitations underscore the need for future research to better refine and accurately quantify these complex dynamics.

## Conclusions

Our findings further illustrate the complex and substantial costs of climate change that lend support for mitigation being less costly than the impacts [e.g., 6, 7]. The anecdotes presented in this paper describe a suite of activities that occur in a band of latitudes across the globe that provide social, cultural, and financial value. With shrinking lake ice, these activities will be relocated northward, turned into other activities (i.e., open water activities), or lost entirely. We acknowledge there is a great amount of uncertainty in this type of exercise around the estimates of ice phenology [78], forecasting inflation/depreciation (for a discussion, see S1 File), and generalizing globally. Our study provides the first efforts to quantify the expected changes in spending from declining lake ice. More importantly, within the wide bands of uncertainty, we can undeniably state that spending habits for many communities previously dependent on lake ice will be changing in the coming decades. We estimated a difference of 10% and 33% in lake ice duration between the high and low greenhouse gas emissions scenarios, suggesting a large benefit to take climate action just in lake ice alone. Additionally, if action to mitigate climate change continues to be delayed, the cost of mitigation will continue to rise or cause the greenhouse concentration limit of RCP 2.6 and 6.0 to become untenable [79]. Critically, climate change effects on lake ice will largely be felt by the economies of countries most capable of enacting change to mitigate the impacts. Continued efforts to quantify the economic implications will be crucial throughout the 21st century as countries expand efforts to both mitigate and adapt to climate change.

## Supporting information

S1 Table Examples of spending from lake ice activities in local communities. (PDF)

S2 Table Summary statistics of currently estimated lake ice area and future predicted ice area under three greenhouse gas scenarios (RCPs). Included for each country are the carbon emissions (metric tons per capita), annual freshwater withdrawal (billions of cubic meters of water), country population (millions of people), and the Gross Domestic Product (millions of USD). A three-letter country code is provided that is used in all figures.
(PDF)

S1 File An examination and discussion of uncertainty when estimating the future costs of lake ice loss. (DOCX)
