# Supplementary material for: Declining lake ice in response to climate change can impact spending for local communities
Source: PLoS One. 2024 Jul 5;19(7):e0299937. doi: 10.1371/journal.pone.0299937 (PMC11226110; doi:10.1371/journal.pone.0299937)
Supplement: S1 Table — (PDF) [file pone.0299937.s001.pdf]

Country	Regions/Lakes	Latitude	Longitude	Activity	Economic Value	Currency	Economic Value (in USD)	People attending	Source	Snapshot
Sweden	General			Ice Fishing	$880,000,000.00	USD	$880,000,000.00		Report - Recreational fishing in Sweden 2017	
Canada	Lake Winnipeg, Manitoba	52.77823	-98.0444	Recreational Ice fishing	$419,000,000.00	CAD	$344,093,275.00	> 100,000 people	https://mwf.mb.c	WebsiteSourceID11.png
USA	General			Ice Fishing	$178,000,000.00	USD	$178,000,000.00		US Dept of Interior	
Sweden	Multiple frozen lakes			Car Winter Testing	$163,800,000.00	USD	$163,800,000.00	over 3000	https://www.autc	WebsiteSourceID3.png
Canada	Ottawa	45.42379	-75.6926	Winterlude	$151,700,000.00	CAD	$121,471,499.50	600000	https://ottawaciti	WebsiteSourceID28.pdf
Canada	Ontario			Ice Fishing	$70,000,000.00	CAD	$57,946,000.00		https://www.onta	WebsiteSourceID4.png
Canada	Nova Scotia			Ice Fishing	$62,500,000.00	CAD	$51,730,000.00		https://www.salty	WebsiteSourceID7.png
China	Jinzhou	41.1348	120.9982	Ice Dragon Boat race	$45,000,000.00	USD	$45,000,000.00	4.47 million	https://www.chin	WebsiteSourceID8.png
USA	State of Minnesota			Winter angling activities	$36,500,000.00	USD	$36,500,000.00		https://conservan	WebsiteSourceID5.pdf
Canada	St. Lawrence River, Quebec	46.82222	-71.2172	Ice canoe race, Bonhomme Winter Festival	$38,000,000.00	CAD	$30,325,900.00	1000000	https://dec.canad	WebsiteSourceID25.png
China	Chagan Lake	45.25968	124.207	Ice fishing culture tourism festival	$29,000,000.00	USD	$29,000,000.00	100000	https://www.prne	WebsiteSourceID15.png
Canada	Bay of Quinte	44.14626	-77.2278	Ice Fishing	$30,000,000.00	CAD	$24,830,839.80	300000	https://www.inte	WebsiteSourceID17.png
Canada	Lake Simcoe	44.33345	-79.4892	Ice Fishing	$28,000,000.00	CAD	$23,175,450.48		https://www.fishi	WebsiteSourceID16.png
Switzerland	Lake St. Moritz	46.49337	9.841849	Horse Racing	$20,400,000.00	USD	$20,400,000.00	10000	https://www.cnn	WebsiteSourceID14.png
USA	Devil's lake	48.11328	-99.0943	Recreational Ice Fishing	$20,000,000.00	USD	$20,000,000.00		https://www.thef	WebsiteSourceID22.png
Canada	Ste-Anne-de-la-Perade, Quebec	46.5518	-72.1697	Ice Fishing	$6,000,000.00	CAD	$4,788,300.00	100000	https://www.cbc.	WebsiteSourceID27.pdf
Canada	Lake Louise, Alberta	51.41314	-116.226	Lake Louise Winterstart World Cup	$3,600,000.00	CAD	$2,979,648.00	7700 (over 100 million media audience worldwide)	https://open.albe	WebsiteSourceID10.pdf
USA	Devil's lake	48.11328	-99.0943	Devils Lake Volunteer Fire Department Ice Fishing Derby	$1,300,000.00	USD	$1,300,000.00	> 5000 people	https://www.grar	WebsiteSourceID23.png
Canada	Kingston, Ontario	44.23885	-76.4907	Ice boat championship	$225,000.00	CAD	$186,214.34		https://canadians	WebsiteSourceID19.png
Canada	Wawa lake, Ontario	48.01037	-84.7402	Wawa Ice Fishing Derby	$175,000.00	CAD	$144,846.57	1300	https://www.lake	WebsiteSourceID18.png
Canada	Lac la biche, Alberta	54.81449	-112	Winter Festival of Speed	$150,000.00	CAD	$124,152.00	Over 100	https://letsgooutc	WebsiteSourceID12.png
Canada	Rideau Canal Skateway, Ottawa	45.40364	-75.6807	Ice dragon boat (international)	$100,000.00	CAD	$79,805.00	25000	https://www.iced	WebsiteSourceID26.png
Canada	COl lake, Alberta	54.50749	-110.126	Alberta 55-plus winter games	$69,100.00	CAD	$57,194.00	900	https://www.lake	WebsiteSourceID13.png
USA	Gull Lake (Minnesota)	46.45863	-94.3792	Brainerd Jaycess Ice Fishing Extravaganza	$13,400.00	USD	$13,400.00	1150000	https://www.brai	WebsiteSourceID20.png
USA	Leech Lake	47.16237	-94.6171	International Eelpout Festival in Walker	$12,000.00	USD	$12,000.00		https://www.outc	WebsiteSourceID24.png
Canada	Sylvan Lake, Alberta	52.34668	-114.127	Sylvan Lake polar bear dip	$12,718.00	CAD	$10,526.19		https://www.sylv	WebsiteSourceID9.png
USA	Mille Lacs Lake (Minnesota)	46.23824	-93.7838	Fishing For Ducks Contest	$5,000.00	USD	$5,000.00	150000	https://www.star	WebsiteSourceID21.png
