# Supplementary material for: Declining lake ice in response to climate change can impact spending for local communities
Source: PLoS One. 2024 Jul 5;19(7):e0299937. doi: 10.1371/journal.pone.0299937 (PMC11226110; doi:10.1371/journal.pone.0299937)
Supplement: S2 Table — Included for each country are the carbon emissions (metric tons per capita), annual freshwater withdrawal (billions of cubic meters of water), country population (millions of people), and the Gross Domestic Product (millions of USD). A three-letter country code is provided that is used in all figures. (PDF) [file pone.0299937.s002.pdf]

Country Code	Country	RCP	Current Ice Area (km2)	Future Ice Area (km2)	Difference Ice Area	Carbon Emissions	Freshwater Extraction	GDP ($M USD)	Population (M people)
AFG	Afghanistan	RCP 2.6	15.5	0	-15.5	0.25	20.28	19291.1	38.04
AFG	Afghanistan	RCP 6.0	15.5	0	-15.5	0.25	20.28	19291.1	38.04
AFG	Afghanistan	RCP 8.5	10.98	0	-10.98	0.25	20.28	19291.1	38.04
ALB	Albania	RCP 2.6	24.09	0	-24.09	1.58	1.19	15279.18	2.85
ALB	Albania	RCP 6.0	24.09	0	-24.09	1.58	1.19	15279.18	2.85
ARM	Armenia	RCP 2.6	900.52	797.99	-102.53	1.76	2.87	13672.8	2.96
ARM	Armenia	RCP 6.0	900.52	661.13	-239.38	1.76	2.87	13672.8	2.96
ARM	Armenia	RCP 8.5	885.63	531.03	-354.6	1.76	2.87	13672.8	2.96
AUT	Austria	RCP 2.6	189.65	104.95	-84.7	7.03	3.49	445075	8.88
AUT	Austria	RCP 6.0	189.65	52.7	-136.94	7.03	3.49	445075	8.88
AUT	Austria	RCP 8.5	166.96	15.19	-151.77	7.03	3.49	445075	8.88
AZE	Azerbaijan	RCP 2.6	27.79	0	-27.79	3.86	12.78	48047.65	10.02
AZE	Azerbaijan	RCP 6.0	27.79	0	-27.79	3.86	12.78	48047.65	10.02
AZE	Azerbaijan	RCP 8.5	34.2	0	-34.2	3.86	12.78	48047.65	10.02
BLR	Belarus	RCP 2.6	547.81	332.61	-215.2	6.13	1.4	63080.46	9.47
BLR	Belarus	RCP 6.0	547.81	157	-390.81	6.13	1.4	63080.46	9.47
BLR	Belarus	RCP 8.5	602.97	107.08	-495.89	6.13	1.4	63080.46	9.47
BEL	Belgium	RCP 2.6	32.46	0	-32.46	8.55	3.99	533097	11.48
BEL	Belgium	RCP 6.0	32.46	0	-32.46	8.55	3.99	533097	11.48
BEL	Belgium	RCP 8.5	29.97	0	-29.97	8.55	3.99	533097	11.48
BIH	Bosnia and Herzegovina	RCP 2.6	14	3.5	-10.5	6.45	0.4	20164.19	3.3
BIH	Bosnia and Herzegovina	RCP 6.0	14	3.5	-10.5	6.45	0.4	20164.19	3.3
BIH	Bosnia and Herzegovina	RCP 8.5	10.77	2.15	-8.61	6.45	0.4	20164.19	3.3
BGR	Bulgaria	RCP 2.6	118.71	54.9	-63.81	5.85	5.66	68558.82	6.98
BGR	Bulgaria	RCP 6.0	118.71	18.46	-100.25	5.85	5.66	68558.82	6.98
BGR	Bulgaria	RCP 8.5	111.15	7.85	-103.3	5.85	5.66	68558.82	6.98
CAN	Canada	RCP 2.6	750258.17	732792.2	-17465.97	15.09	35.73	1736430	37.59
CAN	Canada	RCP 6.0	750258.17	719941.9	-30316.27	15.09	35.73	1736430	37.59
CAN	Canada	RCP 8.5	751570.59	696286.75	-55283.84	15.09	35.73	1736430	37.59
CHN	China	RCP 2.6	21598.24	20505.7	-1092.55	7.18	591.8	14279900	1397.72
CHN	China	RCP 6.0	21598.24	19920.58	-1677.67	7.18	591.8	14279900	1397.72
CHN	China	RCP 8.5	21679.4	18207.21	-3472.19	7.18	591.8	14279900	1397.72
HRV	Croatia	RCP 2.6	59.19	11.17	-48.02	4.19	0.67	60752.59	4.07

HRV	Croatia	RCP 6.0	59.19	7.24	-51.95	4.19	0.67	60752.59	4.07
HRV	Croatia	RCP 8.5	44.24	5.96	-38.28	4.19	0.67	60752.59	4.07
CZE	Czech Republic	RCP 2.6	65.76	26.38	-39.37	9.67	1.63	250681	10.67
CZE	Czech Republic	RCP 6.0	65.76	13.26	-52.5	9.67	1.63	250681	10.67
CZE	Czech Republic	RCP 8.5	64.78	5.38	-59.4	9.67	1.63	250681	10.67
DNK	Denmark	RCP 2.6	209.18	150.86	-58.32	5.55	0.74	350104	5.82
DNK	Denmark	RCP 6.0	209.18	90.42	-118.77	5.55	0.74	350104	5.82
DNK	Denmark	RCP 8.5	213.9	69.67	-144.23	5.55	0.74	350104	5.82
EST	Estonia	RCP 2.6	1706.34	1323.29	-383.05	12.61	1.78	31471.1	1.33
EST	Estonia	RCP 6.0	1706.34	412.33	-1294	12.61	1.78	31471.1	1.33
EST	Estonia	RCP 8.5	1766.88	153.99	-1612.9	12.61	1.78	31471.1	1.33
FIN	Finland	RCP 2.6	32376.38	32229.38	-146.99	8.35	6.56	269296	5.52
FIN	Finland	RCP 6.0	32376.38	30673.85	-1702.53	8.35	6.56	269296	5.52
FIN	Finland	RCP 8.5	32386.11	20374.51	-12011.6	8.35	6.56	269296	5.52
FRA	France	RCP 2.6	145.46	0	-145.46		26.44	2715520	67.06
FRA	France	RCP 6.0	145.46	0	-145.46		26.44	2715520	67.06
FRA	France	RCP 8.5	98.46	0	-98.46		26.44	2715520	67.06
DEU	Germany	RCP 2.6	1023.32	621.22	-402.09	8.84	24.44	3861120	83.13
DEU	Germany	RCP 6.0	1023.32	238.7	-784.61	8.84	24.44	3861120	83.13
DEU	Germany	RCP 8.5	973.23	61.01	-912.22	8.84	24.44	3861120	83.13
GRC	Greece	RCP 2.6	151.65	25.95	-125.7	5.79	11.24	209853	10.72
GRC	Greece	RCP 6.0	151.65	12.97	-138.67	5.79	11.24	209853	10.72
GRC	Greece	RCP 8.5	122.71	0	-122.71	5.79	11.24	209853	10.72
GRL	Greenland	RCP 2.6	3164.43	3161.86	-2.56	9.07			0.06
GRL	Greenland	RCP 6.0	3164.43	3159.3	-5.13	9.07			0.06
GRL	Greenland	RCP 8.5	3400.76	3394.45	-6.31	9.07			0.06
HUN	Hungary	RCP 2.6	279.1	77.46	-201.64	4.64	4.5	163469	9.77
HUN	Hungary	RCP 6.0	279.1	55.7	-223.4	4.64	4.5	163469	9.77
HUN	Hungary	RCP 8.5	224.71	19.05	-205.66	4.64	4.5	163469	9.77
ISL	Iceland	RCP 2.6	842.05	713.23	-128.82	6.15	0.29	24188.04	0.36
ISL	Iceland	RCP 6.0	842.05	558.73	-283.33	6.15	0.29	24188.04	0.36
ISL	Iceland	RCP 8.5	882.52	374.36	-508.15	6.15	0.29	24188.04	0.36
IND	India	RCP 2.6	287.83	213.69	-74.15	1.82	647.5	2868930	1366.42
IND	India	RCP 6.0	287.83	213.69	-74.15	1.82	647.5	2868930	1366.42

IND	India	RCP 8.5	304.94	193.7	-111.24	1.82	647.5	2868930	1366.42
IRN	Iran	RCP 2.6	925.65	103.2	-822.44	8.32	92.95		82.91
IRN	Iran	RCP 6.0	925.65	26.27	-899.38	8.32	92.95		82.91
IRN	Iran	RCP 8.5	829.08	0	-829.08	8.32	92.95		82.91
IRL	Ireland	RCP 2.6	75.78	23.39	-52.39	7.93	0.76	388699	4.94
IRL	Ireland	RCP 6.0	75.78	0	-75.78	7.93	0.76	388699	4.94
IRL	Ireland	RCP 8.5	73.21	0	-73.21	7.93	0.76	388699	4.94
ITA	Italy	RCP 2.6	540.92	148.66	-392.25		34.05	2003580	60.3
ITA	Italy	RCP 6.0	540.92	47.09	-493.83		34.05	2003580	60.3
ITA	Italy	RCP 8.5	556.66	11.59	-545.06		34.05	2003580	60.3
JPN	Japan	RCP 2.6	607.69	408.39	-199.3	8.94	81.22	5081770	126.26
JPN	Japan	RCP 6.0	607.69	320.37	-287.32	8.94	81.22	5081770	126.26
JPN	Japan	RCP 8.5	628.67	148.83	-479.84	8.94	81.22	5081770	126.26
KAZ	Kazakhstan	RCP 2.6	26525.36	24184.53	-2340.83	13.89	22.45	181666	18.51
KAZ	Kazakhstan	RCP 6.0	26525.36	21745.46	-4779.9	13.89	22.45	181666	18.51
KAZ	Kazakhstan	RCP 8.5	26860.46	19409.04	-7451.42	13.89	22.45	181666	18.51
LVA	Latvia	RCP 2.6	737.48	536.83	-200.64	3.57	0.18	34102.91	1.91
LVA	Latvia	RCP 6.0	737.48	216.01	-521.47	3.57	0.18	34102.91	1.91
LVA	Latvia	RCP 8.5	761.04	160.39	-600.65	3.57	0.18	34102.91	1.91
LIE	Liechtenstein	RCP 2.6	8.46	8.46	0	1.36			0.04
LIE	Liechtenstein	RCP 6.0	8.46	6.35	-2.12	1.36			0.04
LIE	Liechtenstein	RCP 8.5	9.11	2.6	-6.51	1.36			0.04
LTU	Lithuania	RCP 2.6	917.41	440.07	-477.34	4.52	0.26	54627.41	2.79
LTU	Lithuania	RCP 6.0	917.41	254.94	-662.46	4.52	0.26	54627.41	2.79
LTU	Lithuania	RCP 8.5	943.09	156.89	-786.2	4.52	0.26	54627.41	2.79
MDA	Moldova	RCP 2.6	117.17	48.03	-69.13	1.83	0.84	11968.71	2.66
MDA	Moldova	RCP 6.0	117.17	23.35	-93.81	1.83	0.84	11968.71	2.66
MDA	Moldova	RCP 8.5	109.26	4.66	-104.6	1.83	0.84	11968.71	2.66
MNG	Mongolia	RCP 2.6	8788.75	8788.75	0	8.3	0.46	13996.72	3.23
MNG	Mongolia	RCP 6.0	8788.75	8788.75	0	8.3	0.46	13996.72	3.23
MNG	Mongolia	RCP 8.5	8831.08	8831.08	0	8.3	0.46	13996.72	3.23
NLD	Netherlands	RCP 2.6	79.01	15.79	-63.22	10.03	7.99	907051	17.33
NLD	Netherlands	RCP 6.0	79.01	0	-79.01	10.03	7.99	907051	17.33
NLD	Netherlands	RCP 8.5	72.94	0	-72.94	10.03	7.99	907051	17.33

NOR	Norway	RCP 2.6	7931.47	7631.41	-300.06	7.84	2.69	403336	5.35
NOR	Norway	RCP 6.0	7931.47	6948.17	-983.3	7.84	2.69	403336	5.35
NOR	Norway	RCP 8.5	7975.89	5084.88	-2891	7.84	2.69	403336	5.35
PAK	Pakistan	RCP 2.6	63.53	63.53	0	0.99	200	278222	216.57
PAK	Pakistan	RCP 6.0	63.53	63.53	0	0.99	200	278222	216.57
PAK	Pakistan	RCP 8.5	63.53	29.32	-34.21	0.99	200	278222	216.57
POL	Poland	RCP 2.6	1671.66	744.53	-927.13	7.88	10.08	595858	37.97
POL	Poland	RCP 6.0	1671.66	539.99	-1131.66	7.88	10.08	595858	37.97
POL	Poland	RCP 8.5	1665.22	259.66	-1405.56	7.88	10.08	595858	37.97
ROU	Romania	RCP 2.6	422.76	145.12	-277.63	3.52	6.77	250077	19.36
ROU	Romania	RCP 6.0	422.76	63.24	-359.52	3.52	6.77	250077	19.36
ROU	Romania	RCP 8.5	388.33	15.29	-373.05	3.52	6.77	250077	19.36
RUS	Russian Federation	RCP 2.6	295701.47	290317	-5384.47	12	64.41	1699880	144.37
RUS	Russian Federation	RCP 6.0	295701.47	280908.43	-14793.04	12	64.41	1699880	144.37
RUS	Russian Federation	RCP 8.5	298205.81	252427.03	-45778.77	12	64.41	1699880	144.37
SRB	Serbia	RCP 2.6	5.46	1.36	-4.09	6.41	5.38	51475.02	6.94
SRB	Serbia	RCP 6.0	5.46	0	-5.46	6.41	5.38	51475.02	6.94
SRB	Serbia	RCP 8.5	5.04	0	-5.04	6.41	5.38	51475.02	6.94
ESP	Spain	RCP 2.6	49.53	0	-49.53	5.25	31.22	1393490	47.08
ESP	Spain	RCP 6.0	49.53	0	-49.53	5.25	31.22	1393490	47.08
ESP	Spain	RCP 8.5	26.34	0	-26.34	5.25	31.22	1393490	47.08
SWE	Sweden	RCP 2.6	31922.14	29329.55	-2592.59	4.36	2.38	530884	10.29
SWE	Sweden	RCP 6.0	31922.14	25038.81	-6883.33	4.36	2.38	530884	10.29
SWE	Sweden	RCP 8.5	32212.59	18548.2	-13664.39	4.36	2.38	530884	10.29
CHE	Switzerland	RCP 2.6	295.42	52.44	-242.98	4.12	1.73	703082	8.57
CHE	Switzerland	RCP 6.0	295.42	29.68	-265.73	4.12	1.73	703082	8.57
CHE	Switzerland	RCP 8.5	268.5	16.18	-252.32	4.12	1.73	703082	8.57
TJK	Tajikistan	RCP 2.6	707.51	532.8	-174.71	0.61	10.42	8116.63	9.32
TJK	Tajikistan	RCP 6.0	707.51	496.32	-211.19	0.61	10.42	8116.63	9.32
TJK	Tajikistan	RCP 8.5	698.8	439.44	-259.36	0.61	10.42	8116.63	9.32
TUR	Turkey	RCP 2.6	3811.32	1118.11	-2693.21	4.67	60.01	761425	83.43
TUR	Turkey	RCP 6.0	3811.32	637.55	-3173.76	4.67	60.01	761425	83.43
TUR	Turkey	RCP 8.5	3478.25	148.49	-3329.77	4.67	60.01	761425	83.43
TKM	Turkmenistan	RCP 2.6	343.99	255.91	-88.09	12.47	27.87		5.94

TKM	Turkmenistan	RCP 6.0	343.99	68.54	-275.45	12.47	27.87		5.94
TKM	Turkmenistan	RCP 8.5	333.66	84.36	-249.3	12.47	27.87		5.94
UKR	Ukraine	RCP 2.6	7484.58	3125.08	-4359.5	4.49	8.64	153781	44.39
UKR	Ukraine	RCP 6.0	7484.58	2109.43	-5375.15	4.49	8.64	153781	44.39
UKR	Ukraine	RCP 8.5	7388	1025.26	-6362.74	4.49	8.64	153781	44.39
GBR	United Kingdom	RCP 2.6	199.57	141.38	-58.19	5.78	8.42	2829110	66.83
GBR	United Kingdom	RCP 6.0	199.57	88.69	-110.88	5.78	8.42	2829110	66.83
GBR	United Kingdom	RCP 8.5	189.92	45.22	-144.7	5.78	8.42	2829110	66.83
USA	United States of America	RCP 2.6	181217.54	148972.25	-32245.29	15.5	444.4	21433200	328.24
USA	United States of America	RCP 6.0	181217.54	125363.76	-55853.78	15.5	444.4	21433200	328.24
USA	United States of America	RCP 8.5	177935.61	83163.94	-94771.67	15.5	444.4	21433200	328.24
UZB	Uzbekistan	RCP 2.6	488.88	286.32	-202.56	2.88	58.9	57921.29	33.58
UZB	Uzbekistan	RCP 6.0	488.88	117.57	-371.31	2.88	58.9	57921.29	33.58
UZB	Uzbekistan	RCP 8.5	479.08	125.17	-353.91	2.88	58.9	57921.29	33.58
