# Supplementary material for: Declining lake ice in response to climate change can impact spending for local communities
Source: PLoS One. 2024 Jul 5;19(7):e0299937. doi: 10.1371/journal.pone.0299937 (PMC11226110; doi:10.1371/journal.pone.0299937)
Supplement: S1 File — (DOCX) [file pone.0299937.s003.docx]

**Supplemental S1 – Uncertainty when estimating the future spending associated with lake ice activities**

**Methods**

Estimating the future spending implications of lake ice loss from climate change involves a substantial amount of uncertainty. There is uncertainty surrounding the lake models, climate projections, inflation rates, and human actions in response to decreased lake ice. Providing accurate predictions of monetary losses experienced in the future is therefore challenging. However, we provide some estimates below based on percent decline in lake ice including some discussion of associated uncertainty. Assuming lake ice activities currently return 1.30 billion USD in economic benefits per year, we predict until the end of the century the potential loss based on the percent decline in lake ice duration.

Forecasting discount rates decades into the future results in a substantial amount of uncertainty. This is especially true in the case of interest rates far past the maturity of liquid fixed income instruments. Easy to interpret models based on interpolating the current yield curve are considered state of the art. For instance, the European Insurance and Occupational Pensions Authority (EIOPA) makes use of a Smith–Wilson extrapolation and is the industry standard under Solvency II (e.g., Jørgensen 2018; Gonzalez Sanchez and Rodriguez-Sanchez 2021). We wanted to capture future uncertainty and so requited a stochastic model of spot rates. We used the 1-factor model demonstrated by Balter et al. (2014) as it is a simpler more parsimonious model appropriate to this application than an arbitrage-free 3 factor models consistent with yield curve extrapolation, such as the Nelson-Siegel Method and its descendants (Christensen et al. 2011)^.^

We first calibrated to the US treasury yield curve to estimate the Risk-Neutral model parameters. Then we compared this to the real-world model parameters by fitting to the Effective Federal Funds Rate (McCracken and Ng 2016) after accounting for weekly effects. The shared parameters (i.e., variance) were comparable and the market price of risk consistent with average values found by other authors (Ahmad and Wilmott 2006). The long-term behaviour of the model yields were also comparable to the Ultimate Forward Rate published by EIOPA ([www.eiopa.europa.eu/](http://www.eiopa.europa.eu/)). For these reasons and others, we believed the model was reasonable over long time scales. A word of caution as the spot rate we used could frequently be negative in forecasts. Given the current low interest environment and higher inflation this is not unreasonable in a real sense but is nonetheless unaccounted for explicitly in the model.

**Results and Discussion**

In our economic examples, we estimate an average change in annual spending between $55 ± 5.26 million USD (RCP 2.6), $200 ± 7.8 million (RCP 6.0) and $409 ± 12.9 million (RCP 8.5) (Fig. S1.1). Each of these three averages captures some of the uncertainty around predictions of greenhouse gas emissions over the next few decades. Additionally, the error value included represents the uncertainty around the global circulation models, lake ice models, and discounting rate. By the end of the century, we estimate this will result in cumulative decline in spending of $4.35 (RCP 2.6), $8.9 (RCP 6.0), or $18.7 (RCP 8.5) billion USD from lake ice activities.


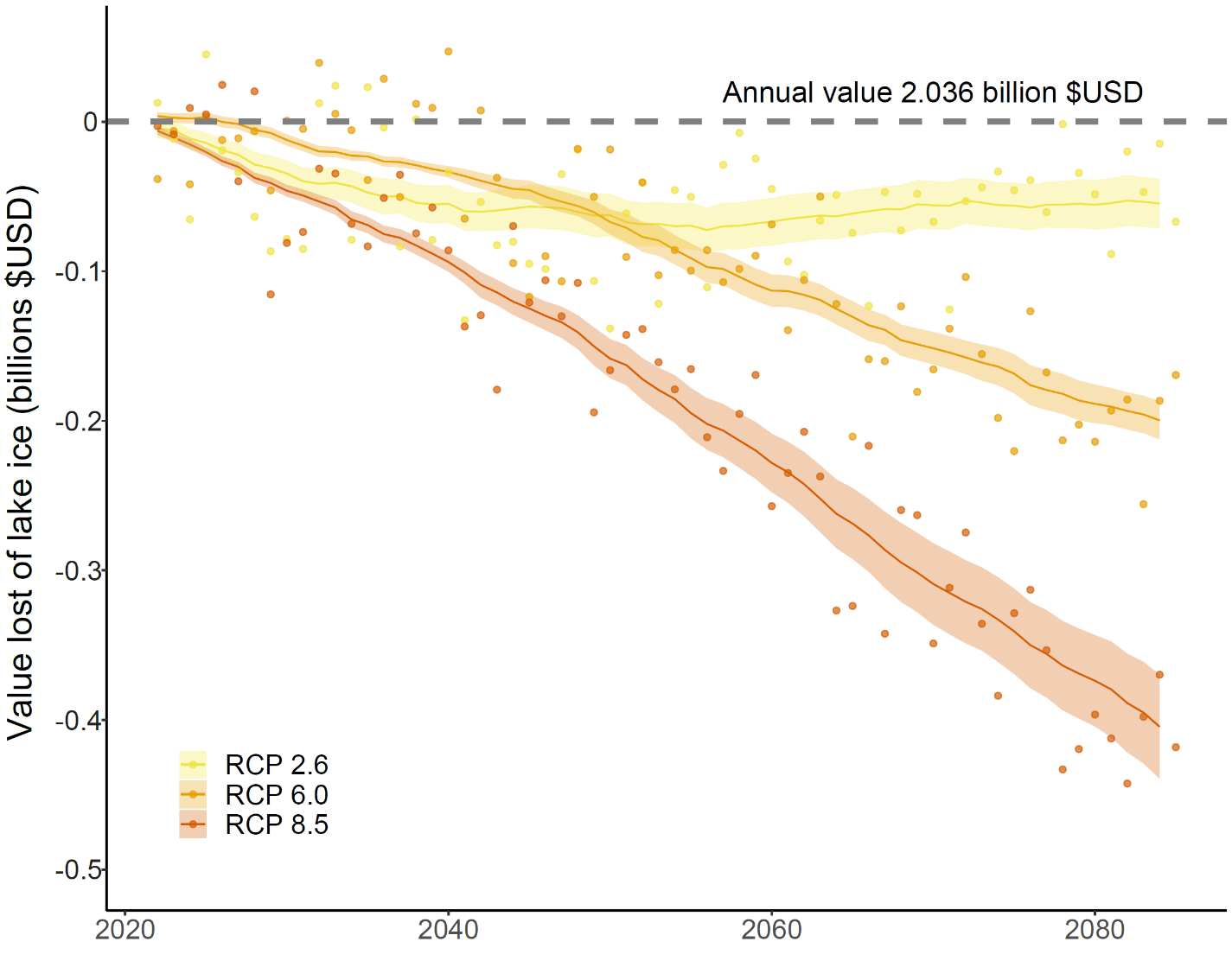


**Figure S1.1:** Assuming a shorter duration of lake ice cover relates to a shorter window for spending (e.g., less time for ice fishing), climate change may cause an average annual monetary loss of between $48 and $396 million (USD) depending on the RCP scenario, translating to a cumulative total of between $2.1 and 16.3 billion (USD) lost by the end of the century. Points represent total annual spending based on percent change in duration of ice cover and solid lines represent a 31-year running average. Estimates of spending until the end of the century have been adjusted based on future values to account for inflation. Lines represent mean model fit and standard error band represents the uncertainty associated among the different GCMs and lake models used to estimate ice phenology.


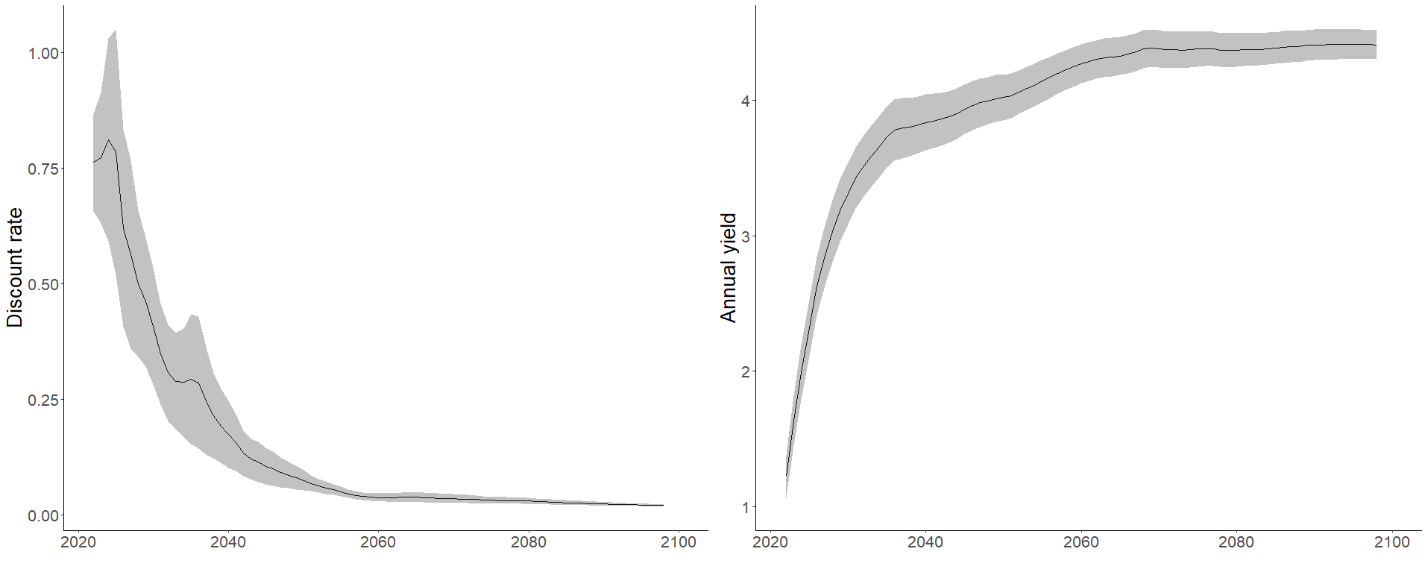


**Figure S1.2:** Projections of annual yield and associated discount rate to be applied to the revenue generated from lake ice activities until 2100. Error band represents uncertainty from 100 simulations of the model. Over the next decade, we predict an increase in the annual yield, but high a high amount of uncertainty in the discount rate, that is expected to stabilize around 4% yield after 2050.

Evaluating the future value of spending generated from lake ice can be generalized into two potential schools of thought. Firstly, there is the belief that the value of lake ice spending should be discounted annually as the valuation of money today will be less in the future. Consequently, the example that we identified which summed to $2.1 billion annually would likely be worth substantially less by 2100. This approach would mean the annual revenue generated from lake ice would closely follow the discount rate curve in Figure S1.2. The second school of though assumes that the value of money generated from lake ice activities would be greater value in the future. For example, if someone were to invest all the money generated from lake ice revenue each year, that money would appreciate based on the interest rate, here assumed to be the projected yield. In this approach, the annual revenue generated would closely follow the yield curve in Figure S1.2. The evaluation and interpretation of these two approaches are extremely different. In the discount-rate approach, spending from lake ice activities are expected to be static and are discounted throughout the century to present day value. In the yield-curve approach, spending is expected to appreciate and are evaluated in the year 2100. Since these approaches have different interpretations, we present both under all three greenhouse gas emission scenarios (Table S1.2).

**Table S1.1:** Evaluations of revenue generated from lake ice discount to present day value and to the year 2100 based on the rates provided in Figure S1.1. We present the mean annual revenue for 2070-2099 and associated standard error. We also include the cumulative sum from 2022-2099. All values are presented in millions of dollars ($USD).

| **RCPs** | **Discount Mean 2070-2099** | **Discount Cumulative Sum** | **Yield Mean 2070-2099** | **Yield Cumulative Sum** |
| --- | --- | --- | --- | --- |
| RCP-2.6 | -1.45 ± 0.15 | -458.11 | -8,323.23 ± 850.6 | -955,900 |
| RCP-6.0 | -5.34 ± 0.18 | -430.64 | -30,671.67 ± 1007.97 | -1,523,796 |
| RCP-8.5 | -10.86 ± 0.23 | -933.1 | -62,459.46 ± 1261.83 | -3,473,753 |

**References**

1. Ahmad, R., and P. Wilmott. 2006. The market price of interest-rate risk: Measuring and modelling fear and greed in the fixed-income markets. Wilmott Mag. 64–70.
2. Balter, A., A. Pelsser, and P. Schotman. 2014. What does a term structure model imply about very long-term discount rates? Available SSRN.
3. Christensen, J. H. E., F. X. Diebold, and G. D. Rudebusch. 2011. The affine arbitrage-free class of Nelson–Siegel term structure models. J. Econom. **164**: 4–20. doi:https://doi.org/10.1016/j.jeconom.2011.02.011
4. Gonzalez Sanchez, M., and S. Rodriguez-Sanchez. 2021. Comparative analysis of interest rate term structures in the Solvency II environment. J. Risk Financ. **22**: 16–33. doi:10.1108/JRF-04-2020-0067
5. Jørgensen, P. L. 2018. An analysis of the Solvency II regulatory framework’s Smith-Wilson model for the term structure of risk-free interest rates. J. Bank. Financ. **97**: 219–237. doi:https://doi.org/10.1016/j.jbankfin.2018.10.001
6. McCracken, M. W., and S. Ng. 2016. FRED-MD: A Monthly Database for Macroeconomic Research. J. Bus. Econ. Stat. **34**: 574–589. doi:10.1080/07350015.2015.1086655
